# Upper extremity deep venous thrombosis prevalence in the NHS Grampian Medical Ambulatory clinic: diagnostic, therapeutic, and prognostic considerations in oncology patients

**DOI:** 10.1007/s11845-021-02775-0

**Published:** 2021-09-13

**Authors:** Stavroula Lila Kastora, Olusegun Oduyoye, Shafaq Mahmood

**Affiliations:** 1 Medical Sciences & Nutrition, University of Aberdeen School of Medicine, Aberdeen, UK; 2 Emergency Care Centre, Aberdeen Royal Infirmary, Foresterhill Campus, Aberdeen, AB25 2ZN UK

**Keywords:** Bioinformatics, Oncology, Prevalence, Survival analysis, Upper extremity deep venous thrombosis

## Abstract

**Introduction:**

Whilst upper extremity deep vein thromboses (UEDVT) account for approximately 5 to 10% of all cases of DVT, rigorous guidelines regarding diagnosis and management of presenting patients remain to be developed. The association of UEDVT with concurrent asymptomatic pulmonary embolism as well as the first presentation of malignancy deems essential rigorous research and clinical guideline development to ensure optimal patient care.

**Methods:**

This retrospective audit study is the first to provide estimates of UEDVT prevalence in the North-East Deanery main hospital centre, Aberdeen Royal Infirmary (ARI).

**Results:**

Of the 605 patients attending the ARI Ambulatory Emergency Care (AEC) clinic with clinical suspicion of UEDVT, 38 (6.2%) had a confirmatory diagnosis. Underlying malignancy, presence of PICC line, and cardiovascular co-morbidities were identified as common confounding factors. Subclavian vein with concurrent extension to primarily the cephalic vein thrombosis was identified as the most commonly thrombosed venous territories. Importantly, oncology patients were found to have poorer survival outcomes following an UEDVT, in comparison to patients with other significant co-morbidities (cardiovascular, chronic renal disease, inflammatory bowel disease): HR 5.814 (95%CI 1.15, 29.25), *p* 0.012. Lastly, genetic associations were drawn between patient genetic status as tested for other co-morbidities and prothrombotic cellular cascades, suggesting rigorous VTE assessment in patients identified with congenital or acquired mutations, namely, in *CALR*, *JAK*, *MSH* 2/6, *MYC*, and *FXN*.

**Conclusions:**

Overall, this study offers the first report of UEDVT presentations in the UK with no restrictions of patient performance status or underlying co-morbidities and provides a rounded clinical picture of patient characteristics, diagnosis, management, and prognostic associations in view of rigorous guideline development.

**Supplementary Information:**

The online version contains supplementary material available at 10.1007/s11845-021-02775-0.

## Introduction

Primary upper extremity deep vein thrombosis (UEDVT) presents a rare clinical entity with an estimated annual incidence of 1 to 2 cases per 100,000 populations. Primary UEDVT, also referred to as Paget-Schroetter syndrome or “effort” thrombosis, is defined as thrombosis of the deep veins draining the upper extremity due to anatomic abnormalities resulting in axillosubclavian compression [1, 2]. This clinical entity typically affects young and physically active individuals presenting as sudden, severe upper extremity pain, and swelling after strenuous upper-body activity. An aggressive therapeutic approach that comprises anticoagulation, catheter-directed thrombolysis, and thoracic outlet decompression present one of the fundamental treatment strategies at relieving acute symptoms and minimizing complications, such as recurrent thromboembolism and post-thrombotic syndrome, in this patient group. On the contrary, secondary or provoked UEDVT presents the majority of upper extremity thrombotic clinical presentations. Secondary UEDVTs primarily occur due to deep vein interventions including central or peripherally inserted catheters (PICC) and pacemaker or defibrillator insertions. Other provoking factors such as prothrombotic states such as inherited or acquired thrombophilia and malignancy have also been identified [3, 4]. Pulmonary embolism (PE) as a secondary thrombotic event following an UEDVT remains a serious complication and accounts for approximately 6% of cases [5] whilst the incidence of asymptomatic PE in patients with UEDVT may range from 3 to 36% [6].

The gold standard of UEDVT management as per causative factor remains obscure, potentially reflecting the rarity of UEDVT as a clinical presentation. For patients with clinically suspected UEDVT, Constans et al. (2008) combined clinical signs and symptoms with risk factors for UEDVT to derive a clinical decision score comparable with those used for lower-extremity DVT [7]. The feasibility and safety of a diagnostic algorithm for UEDVT were evaluated in the ARMOUR study, which was a multicentre prospective study on 406 patients with clinically suspected UEDVT [8].

Furthermore, guidelines on initial diagnosis or treatment options tailored to patient groups and their requirements are still to be developed. The timeline of maintenance of peripherally inserted central catheters after a thrombotic event also remains to be clarified (NICE guidelines) [9]. Lastly, whilst UEDVT has been investigated as a marker of occult malignancy in previous work, the clinical utility of searching for occult cancer in patients presenting with UEDVT remains unclear [10].

The aim of this retrospective audit study was to assess the prevalence of UEDVT in patients visiting the AEC Ambulatory Emergency Clinic (AEC) of the Aberdeen Royal Infirmary between 2015 and 2020, identify causative factors, and highlight commonalities in medical and interventional management tailored to patient presentation. Furthermore, we explored the potential of UEDVT to act as a prognostic factor of mortality in patients with underlying malignancy and putative genetic associations in this patient group.

Overall, this study offers the first report of upper extremity DVT presentations with no restrictions on patient performance status or underlying co-morbidities. Consequently, this study provides a rounded clinical picture of patient characteristics, diagnosis, management, and prognostic associations in view of rigorous guideline development.

## Materials and methods

### Study design

This is a retrospective audit study. Eligible for inclusion were all patients arriving in the Ambulatory Emergency Care (AEC) clinic with potential UEDVT during 2015–2020 in the Aberdeen Royal Infirmary (NHS Grampian). These patients were identified through filtering the AEC records according to presenting complaint.

### Data collection and definition of study exposure

The following clinical and demographic data were collected from electronic clinical records: date of AEC presentation, age, gender, identification of deceased patients (date of death), BMI, co-morbidities (myocardial, respiratory, congenital and/or haematological conditions, malignancy), PICC or Pacemaker line insertion, date of insertion and date of removal, D-Dimer (requested—yes/no, result), imaging modality (ultrasound (US), Computerised Tomography (CT) Angiogram, Venogram), date of imaging modality, venous territories involved, treatment option (medical, interventional), and, if applicable duration, pharmacological treatment, and category of therapeutic agent. Electronic medical records were also studied for information on the UEDVT causative factors, genetic factors involved, type of therapeutic agent used (active agent), duration of use, recurrent UEDVT presentation, and specialty input according to patient confounding condition. Ethical approval was sought from the Research and Development department of the University of Aberdeen. Given that this study was conducted in the context of an audit, we were advised to register the study under the Quality assessment and improvement records of NHS Grampian database; Project code 5295. All patient identifiers were removed, and numerical series were used to code for each patient.

### Primary and secondary outcomes

Among the primary outcomes of the study was to identify how many patients with potential UL-DVTs presenting in AEC were diagnosed with UEDVT. Another primary outcome involved the identification of primary vs. secondary UEDVTs, causative factors, and demographics. Secondary outcomes involved assessment of compliance with current guidelines regarding UL-DVT diagnosis (D-Dimer and imaging modality selection). UEDVT diagnosis is in oncology vs. patients with other co-morbidities and correlation with worsening survival prognosis.

### Statistical analysis

The coded dataset is available upon request. Statistical analysis was performed using GraphPad Prism (v. 8). Pearson correlation analysis (*R*-values) and statistical significance (*p*-values) are available upon request. GraphPad (GP) statistical significance scale was employed to obtain four decimal significance values. Pearson correlation matrix was employed to compute the correlation coefficient for each pair of variables, regardless of other variables. Data were assumed to have been sampled from Gaussian distribution for continuous parameters; thus, the Pearson correlation test was employed. Survival analysis was conducted by Log-rank (Mantel-Cox) and Gehan-Breslow-Wilcoxon test.

STRING analysis of protein association as shown in Fig. 5, was performed with STRING: Functional Protein Association Networks (https://string-db.org) online platform. The addition of connective nodes was performed up to the first degree. Data were retrieved from text mining, experiments, and databases. Edge colours represented the protein–protein interactions as identified experimentally (pink, light blue) or predicted in silico (green-gene neighbourhood, red-gene fusions, blue-gene co-occurrence). Yellow edges indicate associations as identified via text mining whilst black edges signify co-expression.

## Results

### Study participants

A total of 605 patients were identified via the AEC records as potentially eligible for inclusion. The highest arrival peak was identified in 2018 [*N* 197]. The confirmed UEDVT diagnoses per year ranged from 3 in 2020 (min) to 10 in 2016 (max) (mean 6.16; SD 2.48) (Fig. S1).

### Patient demographics and clinical details

**Fig. 1:**
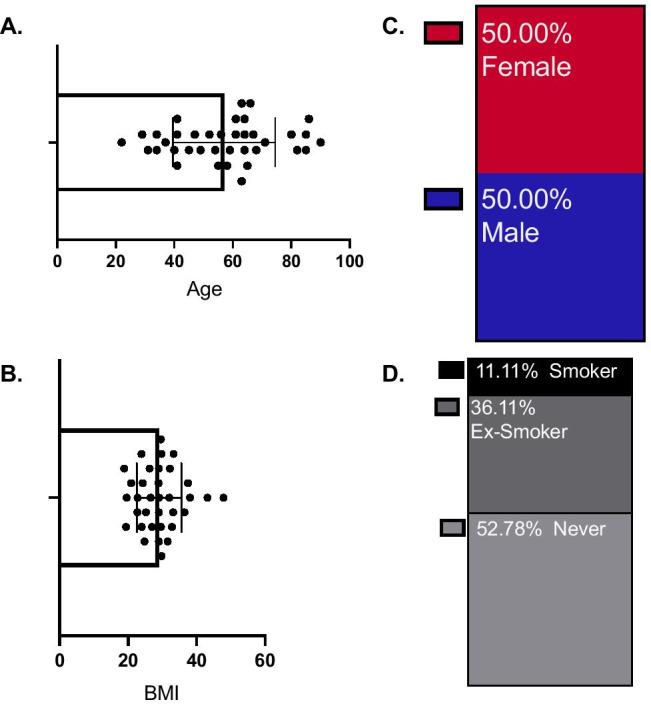
Patient demographics (*N* 38). **A** Age with median and SD. **B** BMI with mean and SD. **C** Gender. **D** Smoking status. The figure was generated using GraphPad Prism V. 8. BMI (body mass index), SD (standard deviation)

**Fig. 2:**
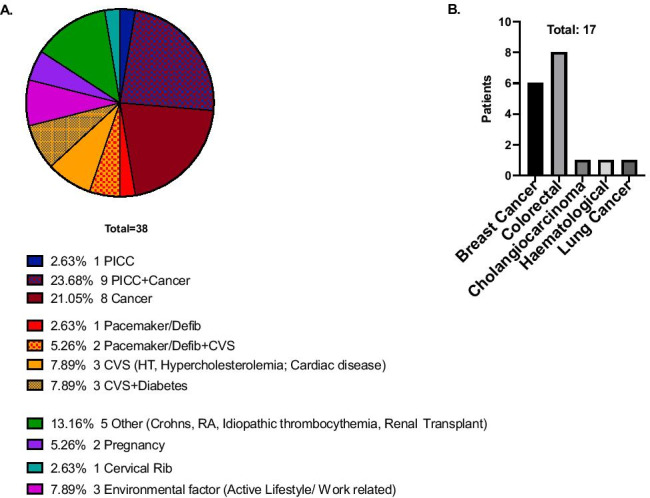
Patient comorbidities. Pie chart of risk factors as identified in our cohort for developing UEDVT **A** and bar chart representation of malignancy type distribution in selected patient cohort **B**. The figure was generated using GraphPad Prism V. 8. *Peripherally inserted central catheter (PICC), T2DM (type 2 diabetes mellitus), Defib (defibrillator), CVS (Cardiovascular co-morbidities), HT (hypertension), RA (rheumatoid arthritis)

A total of 38 patients had a confirmed UEDVT, 6.2% of total UEDVT potential presentations (*N* 605). Other diagnoses included cellulitis (67%; *N* 405), trauma (24%, *N* 145), lymphoedema from known radiotherapy (3%, 17). The median age at presentation was 57, and mean BMI was 29.08 (Fig. 1A, B). A total of 50% of the patients (*N* 19) were male (Fig. 1C). In our patient cohort, 11.11% were smokers, and 6.11% were ex-smokers (Fig. 1D). The majority of patients had a significant underlying co-morbidity. Namely, 24.73% had an active malignancy, out of which 23.68% required a PICC line insertion (Fig. 2A). Another significant risk factor was attributed to cardiovascular co-morbidities (including cardiomyopathy, previous myocardial infarction, hypertension, and familial hypercholesterolemia). A total of 21.05% [*N* 8] were diagnosed with one of the aforementioned cardiovascular illnesses, of which 5.26% [*N* 2] required a pacemaker or defibrillator insertion (Fig. 2A). Only in 7.89% [*N* 3] of the patients, active lifestyle, and increased physical activity (e.g., Paget-Schroetter) were identified as a provoking factor of UEDVT (Fig. 2A).

### Diagnostic modalities

**Fig. 3:**
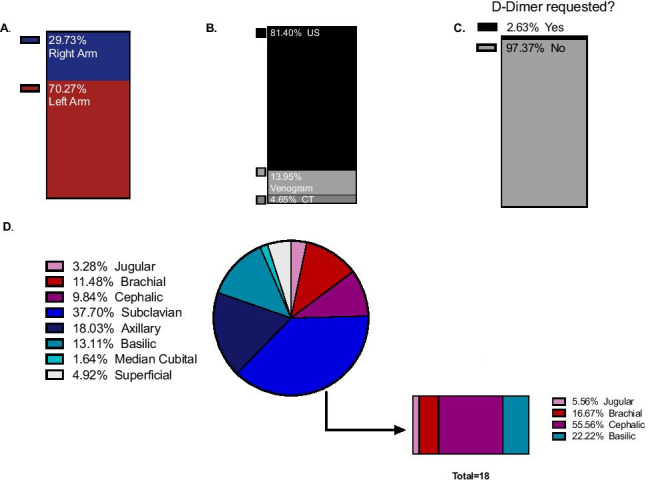
DVT characteristics and incidence (*N* = 39). **A** Arm affected. **B** Treatment. **C** Diagnostic imaging modality. **D** Vein affected and subgroup analysis of subclavian DVTs extending to further vein territories. The figure was generated using GraphPad Prism V. 8. US (ultrasound), CT (computed tomography angiogram)

In our patient cohort, arm involvement was in 29.73% of the presentations right and 70.27% left arm (Fig. 3A). Ultrasound was the imaging modality of choice according to national and international guidelines with 81.40% of the patients being diagnosed solely by ultrasound (Fig. 3B). Only 3 patients were primarily assessed with venogram, among which one required a further CT angiogram (Fig. 3B). This patient was diagnosed with cervical rib resulting in venous malformation. In almost for the entirety of our dataset (97.37%), a D-Dimer was not requested, in compliance with the NICE guidelines (Fig. 3C) [9]. Analysis of venous territory involvement as per radiological report indicated that the subclavian was the primarily affected deep vein (37.7%), followed by the axillary (18.03%) and the basilic (13.11%) vein (Fig. 3D). Subclavian thrombosis with concurrent extension to other venous territories occurred in 81.81% of the cases (18 out of 22) (Fig. 3D). Among the extended territories, cephalic vein thrombosis comprised 55.56% of the concurrent cases (Fig. 3D).

**Fig. 4:**
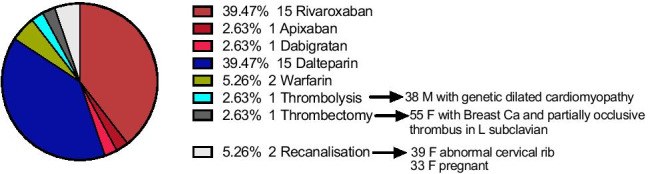
UEDVT medical and interventional management (the figure was generated using GraphPad Prism V. 8. Female (F), breast cancer (Breast Ca), left (L)

Pearson correlation analysis highlighted that the presence of thrombosis in any one of the venous territories often signifies further extension to other; the presence of cervical rib associates with DVTs in all upper extremity venous territories whilst PICC line insertion statistically correlates this basilic thrombotic events (Fig. S2). Patients with co-morbidities, other than malignancy, were mostly treated with DOAC (direct oral anticoagulant) anticoagulation (44.73%) (Fig. 4). Oncology patients or pregnant females were managed with LMWH (39.47%). Two patients who had recurrent DVT (on previous apixaban; rivaroxaban) stepped up to warfarin and apixaban, respectively. Both had further co-morbidities (CVS, T2DM, PICC line insertion). The overall treatment period was 3 months regardless of pharmaceutical agent. Interventional management (thrombolysis, embolectomy) was reserved for only four patients (10.52%), who, in their majority, were significantly younger than the cohort mean (Fig. 4).

### UEDVT as a prognostic factor in oncology patients

**Fig. 5:**
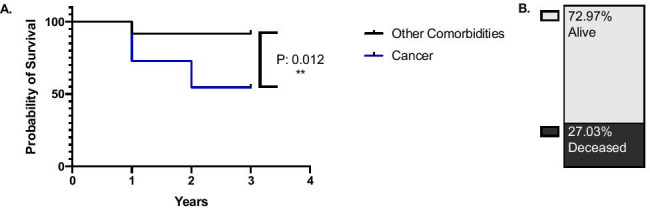
Curve comparison probability of survival after upper extremity DVT in patients with malignancies (colorectal 6; HPB: 1; breast: 1) in comparison to patients with other co-morbidities (cardiovascular; type 2 diabetes mellitus; cervical rib, other). Chi-square 6.310; *p*-value 0.0120; MH log hazard ratio A/B (0.03; 0.86). The figure was generated using GraphPad Prism V. 8

Given that 27.03% of the patients with UEDVT were deceased at the time of the data collection, we explored the potential of UEDVT to act as a poor prognostic factor in specific disease mortality. Curve comparison probability of survival after UEDVT in patients with solid malignancy (breast: 6; colorectal 8; cholangiocarcinoma: 1; lung cancer: 1; non-Hodgkins lymphoma: 1; Fig. 2B) comparison to patients with other co-morbidities (cardiovascular; T2/T1 DM; cervical rib, other) indicated a significant association between the date of UEDVT diagnosis and date of death in oncology patients. The majority of oncology patients (6 out of 17 patients) deceased within the same year or 1 year after the UEDVT diagnosis (Fig. 5). Kaplan-Mayer curves between the two populations significantly differed with *p* value of 0.012: chi-square 6.310 and HR 5.814 [95%CI 1.15, 29.25] (Fig. 5). Overall, this analysis suggests that the probability of mortality post-UEDVT diagnosis with any other comorbidity as a baseline vs. malignancy is significantly increased in oncology patients.

### Genetic considerations

**Fig. 6:**
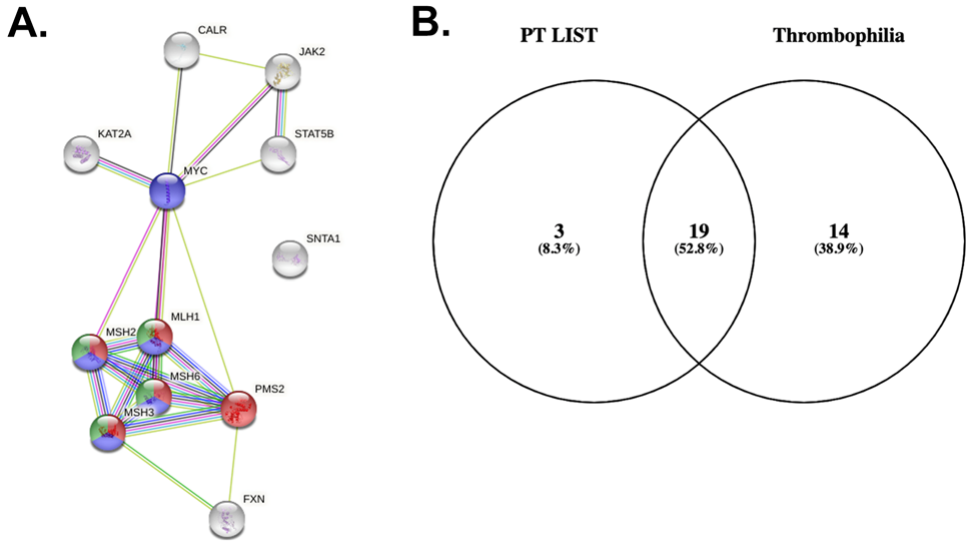
First degree interactome of genetic targets as identified in our patient list **A** and overlap with the first degree interactome routinely screened protein players of thrombophilia screen **B**. PT list (genes identified in patients)

Lastly, we sought to understand whether the genetic background of our patients, as examined for their underlying co-morbidities, played a significant role in their prothrombotic baseline risk. A total of 5 out of the 38 patients had undergone genetic testing and counselling through the medical genetics department of the Aberdeen Royal infirmary. Conditions prompting genetic testing in this group included essential thrombocythemia (*CALR*; *JAK*), colorectal cancer (*MSH 2/6*, *MYC*), Friedrich ataxia (*FXN*), and long QT syndrome (*SNTA1*). The first-degree interactome of these genetic targets was identified through the Protein–Protein Interaction Networks Functional Enrichment Analysis database (STRING V. 11.0b) (Fig. 6A). A similar approach was employed to identify downstream interactome of factors assessed in routine thrombophilia screens (factor V Leiden, prothrombin, antithrombin, protein C, protein S). Comparison of the first degree interactome of encoded proteins as identified in our patient cohort (PT list) and the routine thrombophilia screen (thrombophilia) assessed revealed an overlap of 52.8% (*N* 19) of protein targets (Fig. 6B). This finding supports a solidified biological notion that we rarely see applied in clinical practice, that protein pathways are not restricted to particular phenotypes and pathway interplay is a common scenario. Consequently, whilst specific genetic mutations are identified in the context of explaining a clinical phenotype, it does not imply that they do not extend beyond that phenotype and affect other biological processes, specifically in our case prothrombotic cascades.

## Discussion

In this work, we reviewed 605 patient files that were presented in the AEC, ARI with potential UEDVT between 2015 and 2020. We identified 38 patients with confirmed UEDVT.

Primary UEDVT remains a rare clinical entity and is mostly associated with strenuous upper extremity exercise. Conversely, secondary UEDVT remains a significant complication especially in patients requiring PICCs, with an incidence similar, if not greater than centrally inserted catheters, ranging from 3 to 58% [11]. The incidence of thrombosis in the upper extremity is significantly pronounced in the subclavian vein (18–67%), followed by axillary (5–25%) and the brachial (4–11%) [12]. Concerning PICC-associated DVTs, marked predilection for the left side is observed [12]. This finding potentially reflects that the majority of patients are right-handed, and thus left-sided insertion, in the non-dominant hand is preferred. Among the main complications of UL-DVT, unlike lower limb thromboses, it remains increased mortality, recurrent thromboembolism, and post-thrombotic syndrome. The mortality rate in this patient group can range from 10 to 50%, related mainly to the underlying malignancy, and fatal pulmonary embolism which may contribute to the overall mortality [13]. Our findings, in agreement with recent literature highlighted that use of PICC line, pre-existing cardiovascular conditions, and malignancy, represent independent risk factors of UEDVT [14].

Regarding occult malignancy, UEDVT occurring in the absence of increased activity or thrombophilia and with no identifiable anatomic abnormalities or other contributing benign risk factors should raise concerns of occult malignancy. Up to 25% of patients will be diagnosed with malignancy, within 1 year of UEDVT [15]. Regarding patients with already diagnosed malignancies, UEDVT has been hypothesised to be a poor prognostic factor and may signify increased mortality, a finding that remains to be confirmed. Another clinical consideration for UEDVT presenting patients should be the investigation of an underlying thrombophilic state, and screening for thrombophilia should be included in the initial evaluation [16]. Intriguingly, the overall prevalence of hypercoagulable states in patients with thrombosis of the upper extremities was not significantly higher than in control subjects but was significantly lower than that in patients with deep leg vein thrombosis [17, 18].

In terms of diagnosis, D-Dimer is rarely informative and is not recommended as per NICE guidelines. Consequently, the suggested diagnostic modality is upper extremity Doppler ultrasound, whilst it should be mentioned that contrast venography is more standardised, given the lack of operator subjectivity, and sensitive. A systematic review of 17 studies concluded that compression ultrasonography is an acceptable alternative to standard contrast venography [19]. The summary estimates of the sensitivity of compression, Doppler ultrasound, and Doppler ultrasound with compression were 97, 84, and 81%, respectively, with specificity 96, 94, and 93%, respectively. Of note, regardless of the efficiency of US in UEDVT diagnosis, disadvantages include technician-dependent error and that mural or proximal subclavian thrombi may not be adequately visualised due to acoustic shadowing by the overlying structures [19, 20].

**Fig. 7:**
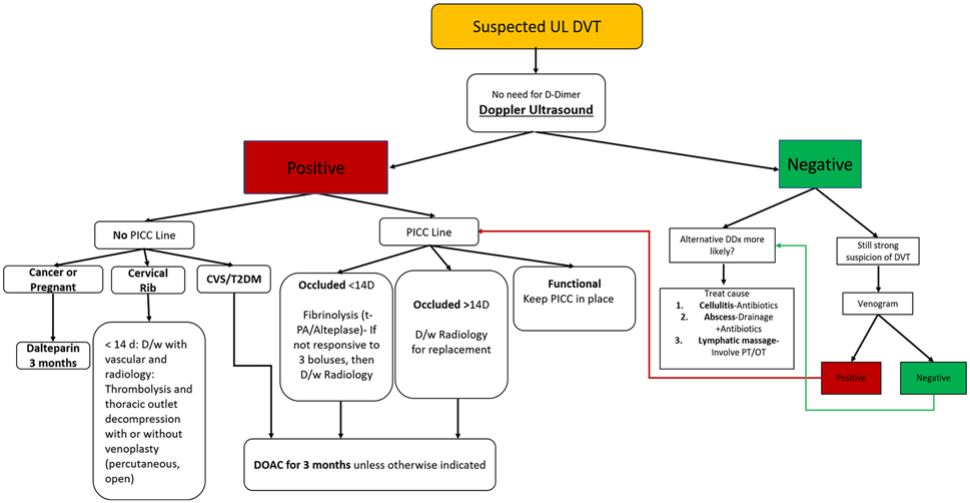
Flow chart of UEDVT patient assessment and downstream medical or surgical management. Peripherally inserted central catheter (PICC), T2DM (type 2 diabetes mellitus), Defib (defibrillator), CVS (cardiovascular co-morbidities), direct oral anticoagulant (DOAC)

Given the rarity of UEDVT, regional and national guidelines regarding work-up and management of affected patients remain obscure. This study aimed to identify the prevalence of diagnosed UEDVTs and confounding risk factors in an ambulatory clinical setting, that serves a large proportion of the rural population in North East Scotland. We further aimed to assess compliance within the limits of current guidelines regarding UEDVT diagnosis (D-Dimer and imaging modality and treatment). We also present a potential flow chart of consistent medical assessment and management of patients with potential UEDVT to ensure cost-effective but, importantly, thorough patient work-up, minimizing misdiagnosis or suboptimal management (Fig. 7).

### Strengths and limitations

Whilst this work is limited by the inherent limitations of a retrospective audit report and may significantly benefit by a broadened base of patients, including medical and surgical, important findings have been raised that warrant investigation in a larger cohort setting. We highlighted that an UEDVT diagnosis in oncology patients signifies worsening survival outcomes when compared to patients with other comorbidities. This finding comes in support with previous hypotheses [12, 14, 15]. Importantly, we also highlighted, that genetic mutations identified in our patient cohort within other disease investigation frameworks, may affect other biological processes, specifically prothrombotic cascades, explaining UEDVT presentations. This result warrants further research to identify whether this is an incidental finding, or it should be incorporated in the baseline clinical considerations regarding medical genetic testing for congenital conditions.

## Conclusion

There is no “one size fits all” approach to the treatment of UEDVT. Tailored care is required given the plethora of underlying factors that may contribute to the presentation, with special consideration to the potential of underlying malignancy. Guidelines regarding the clinical approach and management of the first UEDVT presentation remain to be developed.

## Supplementary Information

Below is the link to the electronic supplementary material.

Supplementary file1 (DOCX 903 KB)
