# Supplementary material for: Upper extremity deep venous thrombosis prevalence in the NHS Grampian Medical Ambulatory clinic: diagnostic, therapeutic, and prognostic considerations in oncology patients
Source: Ir J Med Sci. 2021 Sep 13;191(4):1569–75. doi: 10.1007/s11845-021-02775-0 (PMC9308609; doi:10.1007/s11845-021-02775-0)
Supplement: Supplementary file 1 — Supplementary file1 (DOCX 903 KB) [file 11845_2021_2775_MOESM1_ESM.docx]

**Supplementary material**

Fig. S1. Histogram of arrivals with query UEDVT and diagnoses between 2015-2020.


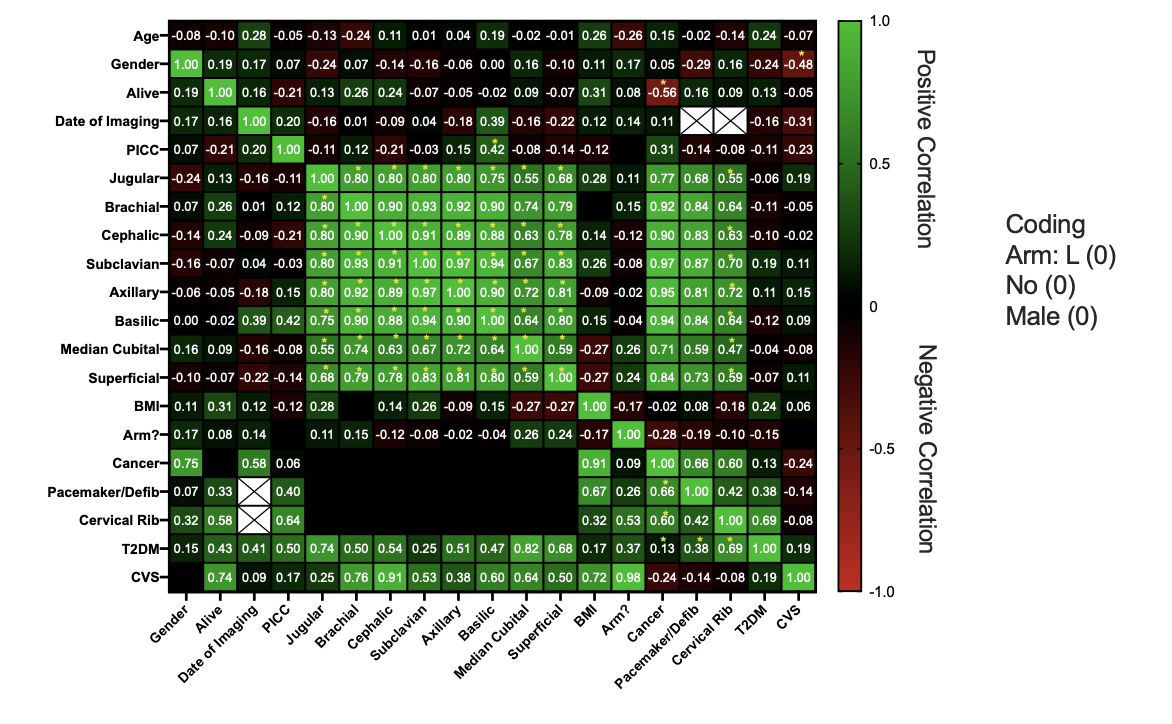


Figure S2. Pearson correlation analysis (r values) of demographic variables and DVT territories. Positive r value (green shades) indicates positive correlation with the respective variable while negative r value (red shades) indicates negative (reverse) correlation. Shades closer to black indicate weak associations either negative or positive. Statistical significance is denoted by *. Peripherally Inserted Central Catheter (PICC), Body Mass Index (BMI), T2DM (Type 2 Diabetes Mellitus), CVS (Cardiovascular co-morbidities.
